# Is MMTV associated with human breast cancer? Maybe, but probably not

**DOI:** 10.1186/s12985-017-0862-x

**Published:** 2017-10-13

**Authors:** Raisa Perzova, Lynn Abbott, Patricia Benz, Steve Landas, Seema Khan, Jordan Glaser, Coleen K. Cunningham, Bernard Poiesz

**Affiliations:** 1 Department of Medicine, Division of Hematology/Oncology, SUNY Upstate Medical University, 750 E. Adams Street, Syracuse, NY 13210 USA; 2 Northwestern University, Feinberg School of Medicine, 250 E Superior, Chicago, IL 60611 USA; 3 Staten Island Hospital, 1408 Richmond Road, Staten Island, NY 10304 USA; 4 Duke University Medical Center, 2301 Erwin Road, Durham, NC 27710 USA

**Keywords:** Mmtv, Human breast cancer, Phylogenetics, Endogenous retroviruses

## Abstract

**Background:**

Conflicting results regarding the association of MMTV with human breast cancer have been reported. Published sequence data have indicated unique MMTV strains in some human samples. However, concerns regarding contamination as a cause of false positive results have persisted.

**Methods:**

We performed PCR assays for MMTV on human breast cancer cell lines and fresh frozen and formalin fixed normal and malignant human breast epithelial samples. Assays were also performed on peripheral blood mononuclear cells from volunteer blood donors and subjects at risk for human retroviral infections. In addition, assays were performed on DNA samples from wild and laboratory mice. Sequencing of MMTV positive samples from both humans and mice were performed and phylogenetically compared.

**Results:**

Using PCR under rigorous conditions to prevent and detect “carryover” contamination, we did detect MMTV DNA in human samples, including breast cancer. However, the results were not consistent and seemed to be an artifact. Further, experiments indicated that the probable source of false positives was murine DNA, containing endogenous MMTV, present in our building. However, comparison of published and, herein, newly described MMTV sequences with published data, indicates that there are some very unique human MMTV sequences in the literature.

**Conclusion:**

While we could not confirm the true presence of MMTV in our human breast cancer subjects, the data indicate that further, perhaps more traditional, retroviral studies are warranted to ascertain whether MMTV might rarely be the cause of human breast cancer.

## Background

Mouse mammary tumor virus (MMTV) is an exogenous retrovirus of murine species which induces mammary carcinomas and T-cell lymphomas in mice after a prolonged latency period. The pathogenesis of the malignancy requires integration of the virus upstream from a host proto-oncogene with subsequent cis-activation of the gene mediated by the promoter and enhancer elements of the viral LTR sequences. The enhancer elements of the LTR are steroid hormone responsive; and ratios of pertinent female hormones in the mouse are known to modulate both viral and host proto-oncogene RNA expression [1].

Unlike simple retroviruses, MMTV contains an additional gene, *sag*, for superantigen, which mediates host infection and tumorigenesis. The Sag protein interacts with major histocompatibility complex class II molecules and the variable region of the β chain of the T-cell receptor. It is capable of inducing a proliferative response in up to 10% of murine T-cells across many νβ families. However, prolonged stimulation induces apoptosis and ultimately loss of the responding cell. This biphasic response also leads to activation of B-lymphocytes by the stimulated T-cells. In early MMTV infection the virus is transmitted via breast milk and B-lymphocytes, activated indirectly by Sag, become the preferred targets in the gut of the newborn mouse. Both B- and T-lymphocytes eventually transport the virus to the mammary epithelium. Ultimately, T-cell depletion blunts the host immune response to the virus and the induced mammary carcinoma [2].

Two forms of MMTV have been described, endogenous and exogenous. The presence of endogenous MMTV DNA varies among wild mice, with some being completely free of MMTV, but these loci have segregated with inbreeding of laboratory strains of mice. The data indicate that there have been multiple infections into murine germ lines over prolonged periods of time [3]. Exogenous MMTV has been found in both laboratory and wild mice [4]. Interestingly, there is not a large amount of sequence data regarding either endogenous or exogenous MMTV strains around the planet, but at least 2–4 distinct substrains have been identified [4, 5].

For many years, researchers have argued whether or not MMTV is associated with human breast cancer [6–21]. Many publications have reported on the successful infection of human cells by MMTV, albeit less efficiently than human cells [22–26]. Further, studies indicate that the MMTV receptor in rodent cells is different than in human cells [27]. Over the past two decades this debate has intensified with claims ranging from a high to no prevalence rates being described by conflicting reports. We decided to investigate this issue ourselves by performing PCR assays under rigorous conditions to prevent and detect “carryover” contamination. We also sought to sequence selected human mammary tumor virus DNA detected and to evaluate MMTV DNA sequences found in wild mice in our area and laboratory strains of mice utilized in our institution.

## Methods

### Subjects

In an IRB-approved archival study we obtained peripheral blood mononuclear cells (PBMC) from 65 subjects deemed to be at risk for retroviral infections [namely, 18 mothers of HIV-1 infected children and 47 HIV-1, HIV-2, HTLV-1 and/or HTLV-2 infected intravenous drug users (IVDU)], and from 100 volunteer blood donors (VBD). We also obtained 66 samples of formalin fixed paraffin-embedded (FFPE) or snap-frozen (SF) biopsies of breast tissue: 10 SF breast cancer, 9 SF fibrocystic breast, 19 FFPE breast cancer, 19 FFPE fibrocystic breast, and 9 FFPE breast reduction samples. The above samples were collected over several years from multiple clinics and operating rooms at Upstate Medical University and Staten Island Hospital. The breast cancer specimens were originally processed in the Pathology Department at Upstate Medical University. All pre-PCR processing was done by one person, in one room, at Upstate, while all PCR and post-PCR work was done by a different person, in a different room, at Upstate.

### Human cell lines

We examined 20 breast cancer cell lines (HCC1419, HCC1569, HCC1500, HCC70, MDA-MB-453, HCC1143, HCC1187, BT474, HCC1395, HCC1428, BT-20, T-47D, MDA-MB-468, HCC38, HCC1954, HCC2218, HCC1599, HCC1937, ZR-75-1, MCF7, obtained from ATCC); 15 malignant T-lymphocyte cell lines (HUT78, HUT102, MOT, UMC 11B, CEM, JK, GA, JS, JC, MOLT4, MT2, UMC ATL-20, NIH CTCL11, SLB-1, MLA144), 10 EBV-transformed B lymphocyte lines (HCC38BL, HCC1143BL, HCC1187BL, HCC1395BL, HCC1428BL, HCC1599BL, and HCC1937BL, obtained from ATCC and UMC402-EBV45, UMC406-EBV51, and UMC412-EBV54 (a kind gift of Steve Graziano), and 5 non-lymphoid malignant cell lines [one sarcoma (HOS); three brain tumors (JON, ROS and BAIRD, a kind gift of Gregory Canute), and one malignant histiocytic lymphoma (ALGR)].

### Rodent tissues and DNA

In a CHUA approved component of the study, DNA for analyses was extracted from the spleens, livers and tails of 18 wild mice cadavers caught in Upstate New York. DNA of laboratory strains and the wild strains, *Mus musculus molassinus, Mus musculus castaneus, Mus spretus, Mus pahari, Mus caroli, WSB/Eij,* and *Czech II/EI* were obtained from Jackson Laboratory, Bar Harbor, ME. DNA was extracted from the livers, spleens and/or tails of laboratory strains of one C3H, one Balb C, one SWR, one NZB, ten C57 Bl6 and ten C57 Bl10 mice. Analyses were also done on DNA in our laboratory from a gerbil, rabbit, rat, squirrel and vole. DNA was extracted from the MMTV infected C3H mammary tumor cell line, Mm5MT (ATCC).

### PCR studies

Pre-PCR work and post-PCR analyses were done by different laboratory personnel in different labs with separate ventilation systems, so as to prevent contamination by previously amplified DNA. DNA was extracted from the various human and murine samples listed above, as previously described [28]. Human studies were done antecedent to rodent studies. Human samples were tested for human β-globin DNA, as previously published [29]. Only samples that tested positive for human β-globin at 0.1 μg and 1.0 μg of DNA input were deemed suitable for subsequent retroviral DNA analyses (i.e. they contained amplifiable human DNA).

Similarly, to check for the presence of suitable amplifiable murine DNA, the murine samples were amplified with a rodent tropomyosin primer pair/probe set. The forward primer MTmF1 (5′-ATG GAC GCC ATC AAG AAG AAG ATG-3′), reverse primer MTmR1 (5′-GCA GAC CTG CTG GCT CCG-3′) and probe MTp (5′-CTC GAC AAG GAG AAC GCC TTG GAT CGA GCT GAG CAA GC-3′) were used. These same primers and probe were used to assess for the contamination of the human samples with rodent DNA.

**Fig. 1:**
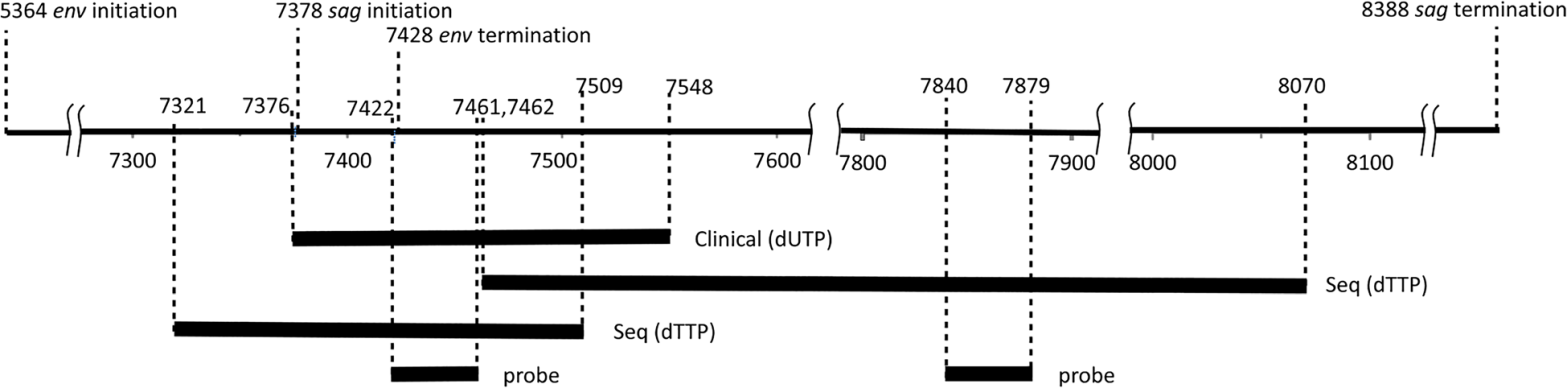
Schematic of the location of the amplicons and probes within the MMTV DNA sequence (GenBank accession number: K01788). Base numbers are as indicated. The vertical wavy lines indicate interruptions in the sequence for display convenience. The locations within the env and sag genes are shown. The clinical amplification utilized dUTP as a substrate and the sequencing amplification utilized dTTP as a substrate. The clinical primers would not amplify the sequencing amplicon and vice versa

DNA was analyzed for the presence of MMTV *env* sequences using viral-specific primers that contained “signature” non-human, non-murine, non-viral (NHNMNV) sequences at their 5′ termini. Because only synthetic amplified DNA would contain them, signature primer pairs, comprised of just the (NHNMNV) sequences, can be subsequently used to determine if a positive result originated from the target viral sequence or from “carryover” contamination [30]. The “signature” primers were forward 5′-TAC GAG CTC GCG AAT TCA TGA T-3′ and reverse 5′-ACA GGT ACC TGC AGA TCT AG-3′. The MMTV *env* primer that contained “signatures” at the 5′- termini were forward BR-7376F 5′-TAC GAG CTC GCG AAT TCA TGA TCC AGA TCG CCT TTA AGA AGG-3′ and reverse BR-7548R 5′-ACA GGT ACC TGC AGA TCT AGT AAT CTG ATC TGA CTG ATC TAC ACT-3′. The probe for amplified MMTV *env* DNA was BR-7422pr 5′-GCT CCT CCA CGG TGG TTG CCT TGC GCC TTC CCT GAC CAG G-3′. This primer pair/probe set was termed the “clinical” detection set (Fig. 1).

MMTV PCR assays were conducted under varying permutations. Optimal conditions for the MMTV PCR were: 1 cycle at 94 °C for 5 min., 45 cycles at 94 °C for 30 s., 55 °C for 30 s., and 72 °C for 45 s., followed by a final extension of 72 °C for 5 min. The 50 μl PCR mixture contained 1 μg or 0.5 μg of target DNA, 200 mM of primer, 200 mM dNTPs, 2.5 mM MgCl_2_, 1 U Taq polymerase. dUTP rather than dTTP was used as a substrate, and all samples were pre-sterilized with uracil-N-glycosylase to prevent carryover contamination [31]. PCR products were electrophoresed into 2% agarose gels containing ethidium bromide and visualized by epi-illumination with UV light. The amplified DNAs were further analyzed, as previously described, by Southern blot hybridization using the above probes [28]. The assay was sensitive to one copy of input MMTV DNA in a Poisson distribution.

Human samples positive for MMTV DNA were reanalyzed with “signature primers” as previously described, to ascertain whether “carryover” contamination could explain the result [30].

The copy number of MMTV *env* DNA was calculated by comparing the amplification results to those of a serial dilution of Mm5MT cells. The relative copy numbers of MMTV *env* DNA in a Mm5MT cell was determined by making serial 10 fold dilutions of Mm5MT cells in the human T-cell line HUT 78 DNA. Replicates were analyzed via MMTV *env* PCR and a Poisson calculation was performed on the results.

### DNA sequencing

Amplicons of MMTV *env-pol* DNA using the primers described below were ligated into TOPO-TA (Invitrogen), transfected into *E. coli* on agar plates, and grown o/n at 37 °C (TOP 10 cells; Invitrogen). Positive colonies were identified by blotting and probing with the MMTV specific sequences above. They were then grown o/n in LB broth. DNA was extracted using QI prep Spin Miniprep Kit (Qiagen Sciences, Germantown, MD) and then sent to the Cornell University Life Sciences Core Laboratories Center (Ithaca, NY) for DNA sequencing using overlapping primers: BR-7321F-5′-CCA ATA CAA AAC TGG TCC CTA-3′ and BR-7507R-AAA TCC CAA AGT AAC CCA AGG-3′, BR-7462F-5′-GGG TGA GTT TTT CTC CAA AAG G-3′ and BR-8070R-5′-AAT CAA AGC AGA TAT GCC CAG-3’ [32]. The respective primers used were termed the “sequencing” primers, and dTTP rather than dUTP was used to generate these amplicons (Fig. 1).

### Phylogenetic analyses

Newly obtained MMTV and human mammary tumor virus (HMTV) DNA sequences and those from databases or published papers were aligned with the *env/pol* regions corresponding to the region amplified by the above PCR conditions (GenBank accession nos: D16249, AY152721, AF228552, KO1788, KO0556, KU184587-KU184607, AY152722, M15122, M22028, M29600, AF243039, AF228551, X64553) [9, 33, 34]. Homologous sequences from the MMTV-related human endogenous HERV-K family of retroviruses served as an outgroup (GenBank accession nos: AF020092, AF012336, AF164612, AF164614, AF164615, AF164610, M14123, AF164613, AF164609, AF164611). Alignments and phylogenetic trees were produced using pileup (Accelrys GCG Seq Web version 3.01 and GCG installed on a UNIX host system). Phylogenetic analysis of the sequences was carried out by using an algorithm for construction of neighbor-joining (NJ) phylogenetic trees as implemented in the phylogeny inference package PHLIP version 3.57C [35]. NJ analysis included 100 resamplings of aligned sequences using the SEQBOOT program. The NJ trees were generated by computing the distance matrixes (Kimura distances, two-parameter model) from bootstrapped sequence data using DND DIST for nucleic acids followed by the Neighbor Program (outgroup rooting and randomized input order options “on”). The majority rule consensus NJ trees were generated using the CONSENSE program. Additionally, the maximum-likelihood method in Puzzle 4.0 package was used [36]. Phylograms were visualized using Tree View [37]. Experimentally derived DNA were subjected to standard nucleotide BLAST searches (http://www.nebi.n/m.nih.gov/BLAST) to define homologies with those deposited at GenBank, European Molecular Biology Laboratory and the DNA database of Japan.

### Statistics

Differences in the prevalence of MMTV sequences in various populations were analyzed using Fisher’s t-test [38].

## Results

### PCR analyses

**Fig. 2:**
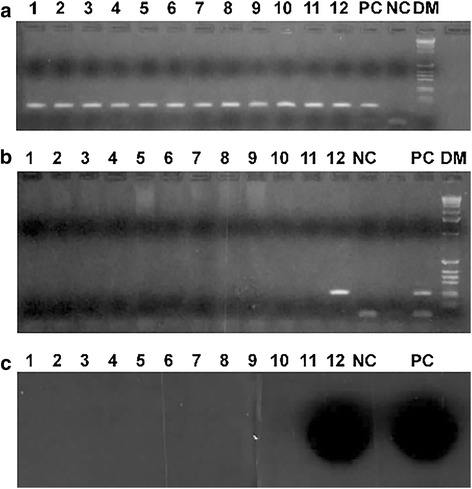
Detection of MMTV DNA in human benign and breast cancer specimens. **a** and **b** are ethidium bromide stained gels, and **c** is a Southern hybridization using the MMTV “clinical” primers and probe. NC is no DNA target added; PC is the positive control containing 100 copies of MMTV DNA extracted from Mm5Mt cells; and DM is the DNA markers. Lanes 4, 8 and 9 are benign breast tissue samples, while lanes 1, 2, 3, 5, 6, 7, 10, 11 and 12 are malignant breast tissue samples. Lane 12 is sample 40,412

Figure 2 and Table 1 show the initial MMTV *env* DNA PCR results using the “clinical” primer pair/probe system on various human samples. As can be seen in Table 1, a number of positive samples were detected. There were 10 out of 86 (11.6%) positive breast samples and 9 out of 195 (4.6%) positive non-breast samples (*p* = 0.031). There were 6 out of 37 (16.2%) positive normal breast samples and 4 out of 49 (8.2%) positive breast cancer specimens (*p* = 0.249). The positive samples were re-amplified with “signature” primer pairs and all were negative indicating the absence of “carry over” contamination. Interestingly, save for one frozen human breast cancer sample (40412) in which the MMTV *env* copy number was ≥1500 copies per μg of human DNA (Fig. 2), all of the other positive human samples had low copy numbers - on average 10 copies/μg of human DNA (data not shown). Because a retroviral-caused cancer, including murine breast carcinoma caused by MMTV insertional mutagenesis and human T-cell lymphoma/leukemia virus which acts in *trans*, usually have at least one copy of integrated provirus per cancer cell (i.e. 150,000 copies/μg tumor DNA) this observation made it unlikely that the MMTV detected in most of the human breast cancers could have been the cause of those breast cancers. This observation and the fact that none of the samples in the retroviral risk group nor breast cancer cell lines were positive also raised the possibility of an artifactual cause of positive results.

**Table 1:** Prevalence of MMTV *env* sequences in various human samples

	MMTV *env*DNA PCR results		
Sample	# tested	# positive	(% positive)
Retroviral risk group	65	0	(0%)
VBD PBMC	100	9	(9%)
Frozen breast Ca	10	1	(10%)
Frozen fibrocystic breast	9	2	(22%)
FFPE breast Ca	19	3	(16%)
FFPE breast fibrocystic	19	2	(10%)
FFPE breast reduction	9	2	(22%)
Breast Ca cell lines	20	0	(0%)
Malignant lymphocyte cell lines	15	0	(0%)
EBV transformed B lymphocyte cell lines	10	0	(0%)
Non-lymphoid malignant cell lines ^a^	5	0	(0%)

^a^ 1 sarcoma, 3 primary brain and 1 histiocytic lymphoma

The MMTV tropomyosin primer pair/probe set gave positive results in all murine, gerbil and rat samples and negative results in the rabbit, squirrel and vole samples. Hence, they could detect DNA from a subset of the Muridine Family of rodents. A possible explanation for a false positive human sample could be contamination with naturally infected (either endogenous or exogenous) MMTV positive rodent DNA. Using this assay, we tested the above MMTV positive human samples for the presence of rodent tropomyosin DNA. A minority of these were positive on any given day that we tested, including aliquots to which we had not added any DNA sample (ie. primer only controls). (A representative experiment is shown in Table 2). The human breast cancer sample (40412) that had the high MMTV copy number was also positive for rodent DNA (Table 2).

**Table 2:** Prevalence of rodent tropomyosin DNA in human samples previously positive for MMTV DNA

Diagnosis	# tested	# Mouse Tropomyosin DNA positive (%)
VBD	9	0 (0%)
Breast cancer	4	2 (50%)
Benign breast	2	1 (50%)
Primer pair only (i.e. no DNA)	79	9 (11%)

**Fig. 3:**
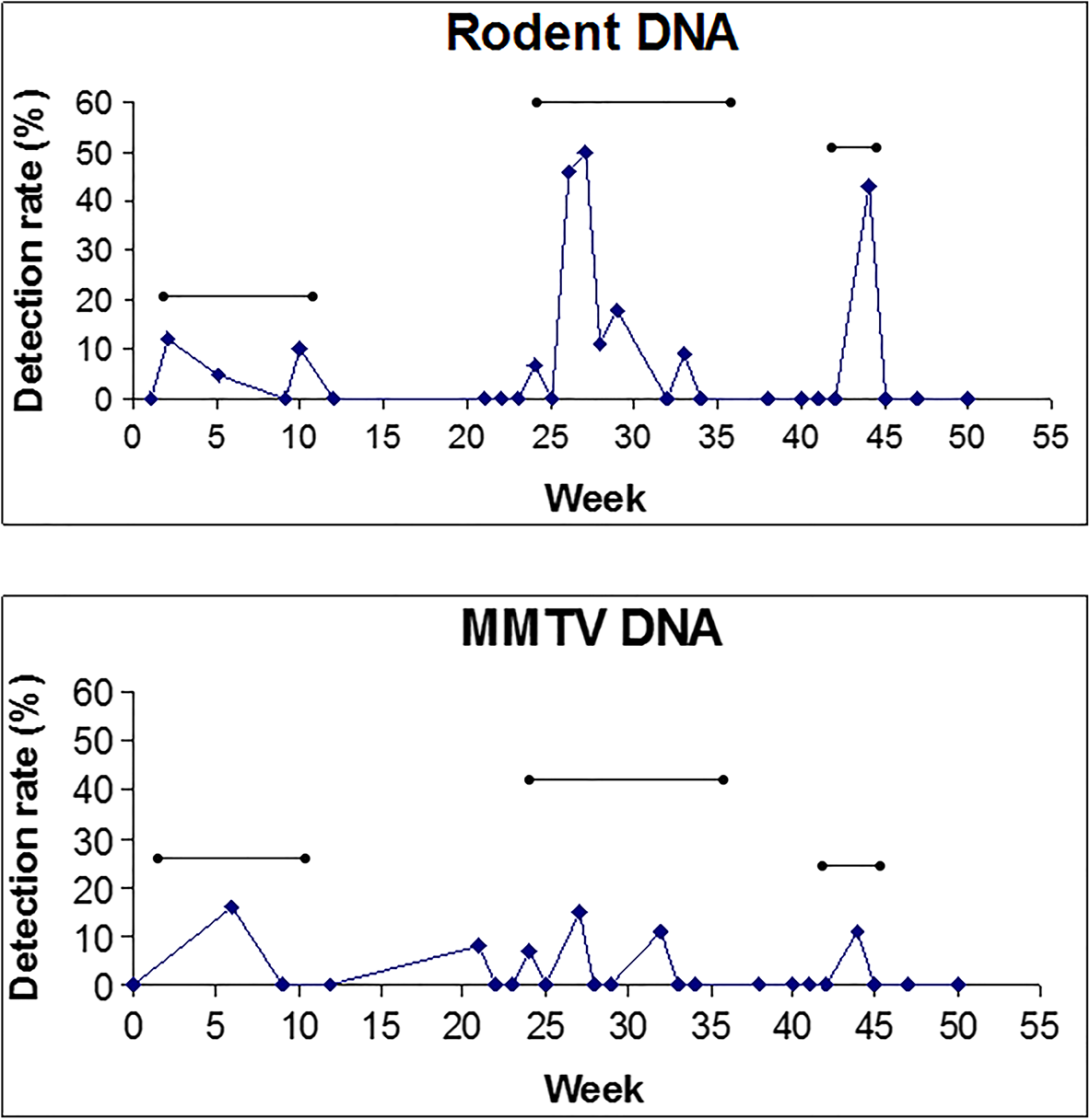
Detection rates of either rodent DNA or MMTV DNA at different time points over a year. The •──• lines indicate periods of time in which major construction projects took place in our building

Theorizing that a source of contaminating rodent DNA could be rodents living, dying and/or being dissected in our own building, we decided to episodically test for rodent DNA and MMTV DNA over a year’s time and correlate that with three periods of construction in our building. As can be seen in Fig. 3, there was a high correlation between these periods and the detection of both rodent and MMTV DNA.

Table 3 shows the MMTV *env* DNA prevalence in various wild mice and inbred strains of mice. All laboratory mice and 11 of 18 wild Upstate New York mice were positive. Using the criteria that, if DNA from the tail, liver, and spleen were all positive for MMTV, we would assume an endogenous infection, four of the 11 wild Upstate New York positive mice were deemed endogenously infected and 7 exogenously infected. All of the samples from the laboratory animals were positive suggesting that they were endogenously infected. Of the samples deemed to be endogenously infected, all had high MMTV DNA copy numbers per μg of sample DNA (data not shown).

**Table 3:** Prevalence of MMTV *env* sequences in various murine samples

Sample			MMTV *env*DNA PCR results		
Species	Strain	Country/State	# tested	# positive	(% positive)
*Mus musculus*	Wild	USA/NY	18	11	(61%)
Mus musculus molassinus	MOLF/EiJ	Japan	1	1	(100%)
Mus musculus castaneus	CASA/RkJ	Thailand	1	1	(100%)
*Mus spretus*	SPRET/EiJ	Spain	1	1	(100%)
*Mus pahari*	Mus pahari/EiJ	Indochina	1	1	(100%)
*Mus caroli*	Mus caroli/EiJ	Thailand	1	1	(100%)
Mus musculus domesticus	WSB/EiJ	USA/MD	1	1	(100%)
Mus musculus	Czech II/EiJ	Czechoslovakia	1	1	(100%)
Mus musculus	C3H	USA/NY	1	1	(100%)
Mus musculus	Balb C	USA/NY	1	1	(100%)
Mus domesticus	SWR	Switzerland	1	1	(100%)
Mus musculus	NZB	New Zealand	1	1	(100%)
Mus musculus	C57 BL6	USA/NY	10	10	(100%)
Mus musculus	C57 BL10	USA/NY	10	10	(100%)

### Sequencing and phylogenetic analyses

**Fig. 4:**
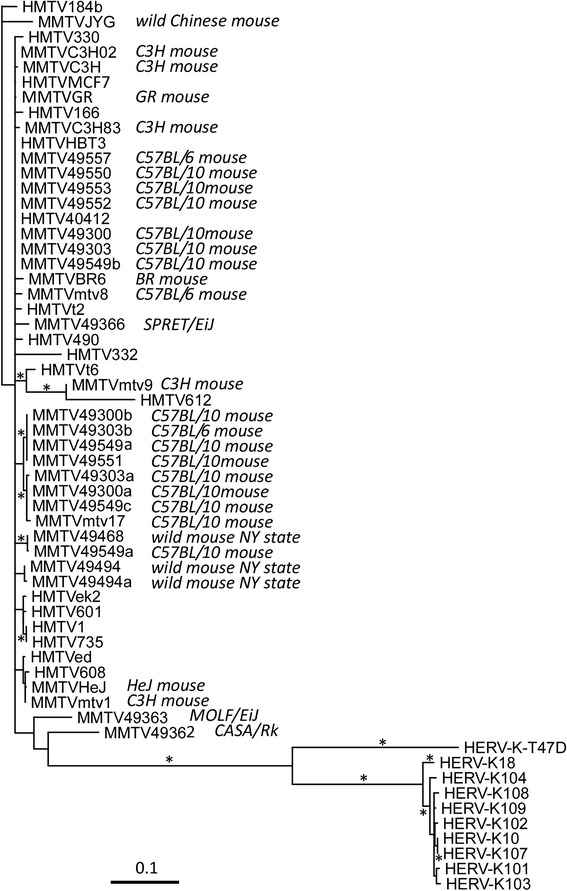
Phylogram demonstrating the relationships among various mouse mammary tumor *env-pol* sequence detected in either mice (MMTV) or human (HMTV) samples. Sequences labeled in the 49,000’s are described herein; all others are from the literature. The mouse strains from which certain sequences were obtained are as indicated. Sequences of human endogenous HERV-K family of retroviruses were used as an outgroup. The symbol (*) indicates branches where the bootstrap value was above 90%. The bar at bottom indicates a distance of 10%

Only one human sample (HMTV 40412) had enough DNA and MMTV copy number to allow for *env* sequencing (GenBank accession #AY152722). We obtained MMTV *env* sequence from one C3H mouse, one C57 Bl 6 mouse, six C57 Bl 10 mice, one SPRET/EiJ mouse, one CASA/RkJ mouse, one MOLF/EiJ mouse, and two wild mice from New York State. Several of these animals had more than one MMTV sequence among the clones analyzed. These new, either human (HMTV) or murine (MMTV), mammary tumor virus sequences were compared phylogenetically to published HMTV and MMTV sequences (Fig. 4). The sequences of the regions chosen as the “clinical” primers and probes in this study were 100% conserved in all published and newly described MMTV and HMTV strains. As seen in Fig. 4, there are at least 5 to 7 major clades of HMTV/MMTV sequences. There were 2 major subclades of MMTV in the C57 Bl 10 mice. Our human strain (HMTV 40412) is identical to an endogenous MMTV sequence found in C57 Bl 10 mice raised in our own facility, suggesting that it could be a contaminant from rodent DNA in our environment. Likewise, several of the published HMTV sequences are very similar or identical to MMTV sequences found in laboratory mice. However, a number of HMTV sequences published by others (eg. HMTV184b, HMTV332, HMTVt6, HMTV612) are quite distinct from any of the MMTV sequences. These HMTV sequences were derived in one lab and presumably collected in metropolitan New York City [34]. The ethnicity of these subjects was not reported.

## Discussion

As mentioned above, there has been considerable debate regarding the association of MMTV and human breast cancer [6–21]. Because many of the more recent publications were based on PCR assays for MMTV DNA, it was quite possible that some positive results were artifactual due to “carryover” contamination of human samples with previously amplified murine MMTV DNA. Unless one takes rigorous precautions to prevent such a phenomenon, this can happen quite easily. In our experiments we went to great lengths to prevent and detect carryover contamination and it is unlikely to be the cause of the positive results in our human samples.

However, our results were not consistent with MMTV as the causative agent of human breast cancer. The results were not skewed toward breast cancer and the MMTV copy numbers detected in the breast cancer specimens were too low to be derived from a monoclonally integrated expanded tumor cell sample. Of course, it is theoretically possible that a human MMTV could cause breast cancer by a mechanism different than that which occurs in mice or other retroviruses. Our experiments indicate that the probable source of false positive HMTV results was contamination with murine DNA containing integrated MMTV DNA. Indeed, the only high copy HMTV breast cancer sample we found was contaminated with rodent DNA and had an MMTV *env* sequence identical to that found in C57 Bl 10 mice present in our facility. We suspect that rodent DNA is present throughout our building’s walls and ventilation systems, most likely as small particulate matter. This possibility has been raised by others but our data confirm it [39]. Indeed, this has been shown to be the probable explanation for the putative association of XMRV with human prostate cancer [40]. Our data would indicate that such contamination is variable on a day to day basis, dependent on the conditions in the laboratory. Our negative data are consistent with the majority of papers on this topic, while only a few labs describe MMTV positive human breast cancer specimens (6–21). To be fair, others have examined MMTV positive human breast samples for the presence of rodent DNA contamination and have not found any [41].

While all of the above refutes claims of an association of MMTV with human breast cancer, it does not explain some of the published HMTV sequence data. We have more than doubled the murine MMTV *env* sequence data available for comparison with published HMTV sequences. As shown in Fig. 4, while many human HMTV sequences are very similar or identical to murine MMTV sequences, there are still quite a few that are very unique (up to 10% divergence). It is unlikely that, barring some major sequencing error or molecular “gymnastics” during PCR amplification, that the murine derived MMTV strains shown in Figure 4 could be the source of these unique HMTV sequences. This raises the question that, if these unusual HMTV sequences are the result of contamination, then contamination by what? These divergent HMTV sequences were all originally derived from one laboratory in New York City [34]. One would not think that MMTV sequences in New York City would be that different from those in Central New York, but it is presumably possible. Of course, there are probably more murine MMTV sequences to be identified throughout the world that, if determined, might resolve this issue.

## Conclusion

In sum, our own data do not make a case for human MMTV infection, and certainly not for an association with human breast cancer. And yet, the unique HMTV sequences described above beg for further inquiry. Is it possible that MMTV is the cause of some rare human breast cancers? Certainly additional sequence data could add to our understanding of this puzzle. But making a case for MMTV being an etiologic agent for human breast cancer will probably require non-PCR based traditional retrovirology techniques such as virus isolation, Southern blot hybridization, and determination of monoclonal integration in tumor cells. This has been done to some degree, but as far as we know, no HMTV isolate has been maintained in continuous culture and fully characterized [42, 43].

## Abbreviations

ATCC: American Type Culture Collection
CHUA: Committee for the Humane use of Animals
*env*: Envelope
FFPE: Formalin fixed paraffin embedded
HERV: Human endogenous retroviruses
HMTV: Human mammary tumor virus
IVDU: Intravenous drug users
LTR: Long terminal repeat
MMTV: Mouse mammary tumor virus
NHNMNV: Non-human non-murine non-viral
PBMC: Peripheral blood mononuclear cells
PCR: polymerase chain reaction
SAC: Superantigen
SF: Snap frozen
VBC: Volunteer blood donors
XMRV: Xenotropic murine leukemia virus-related virus
